# Anatomical and morphometrical approaches on the auditory ossicles in European ground squirrel (*Spermophilus citellus*)

**DOI:** 10.3389/fvets.2025.1609738

**Published:** 2025-08-26

**Authors:** Cristian Olimpiu Martonos, Melissa Kehl, Milos Blagojevic, Ivana Nesic, Florin Gheorghe Stan, Daniel Cocan, Alexandru Ion Gudea

**Affiliations:** ^1^Department of Biomedical Sciences, Ross University School of Veterinary Medicine, Basseterre, Saint Kitts and Nevis; ^2^Faculty of Veterinary Medicine, University of Belgrade, Belgrade, Serbia; ^3^Department of Anatomy, Faculty of Veterinary Medicine, University of Agricultural Sciences and Veterinary Medicine Cluj-Napoca, Cluj-Napoca, Romania; ^4^Faculty of Animal Husbandry and Biotechnologies, University of Agricultural Sciences and Veterinary Medicine Cluj-Napoca, Cluj-Napoca, Romania

**Keywords:** functional anatomy, incus, malleus, middle ear, morphology, morphometry, stapes

## Abstract

**Introduction:**

The study of the middle ear and especially the ossicular chain in different species can bring interesting insights into the biomechanics of hearing. The ground squirrel’s middle ear anatomy has not been studied as thoroughly as its other systems and organs.

**Methods:**

Our study describes the ear ossicles in this little-studied species, providing the morphological and morphometrical characteristics and a series of comparative data, in an attempt to characterise also some functional anatomy of the incus, malleus and stapes.

**Results and discussion:**

The malleus comprises the typical morphological elements, having a round, tuberous head and a very short neck with no bony processes. The manubrium is almost perpendicular to the rotational axis of the ossicle and presents, on its medial surface, the bony process for the insertion of the tensor tympani muscle. Incus is very short and has a deeply incised articular surface for malleus. The lenticular process was identified at the distal part of the long process. The stapes is the smallest ossicle, sheltered in a cavity of the mesotympanic area. It has a very short head, and the stapedial tendon marks its insertion on a visible bony process. An evident surrounding bony ridge marks the elliptic footplate. A comparison of the data related to lever ratio and stapedial surface is also provided based on the collected metrical data.

## Introduction

The *Spermophilus citellus* is a diurnal species of semifossorial rodents, part of a burrow builder group of Sciuridae, which can be found on different continents: Europe, Asia and North America (1–4). European ground squirrels have been reported as an endemic rodent species in Central and Eastern Europe, with an important environmental impact (5). Although during the early 20th century the European ground squirrels were reported as a real pest for European agriculture, a few decades later the number of these specimens had decreased extremely (5). Extermination policies targeting these rodents, combined with an aggressive orientation towards intensive agriculture, have led to the *Spermophilus citellus* being listed on the IUCN Red List of threatened species since 1996 (6, 7). Because the species is situated low in the trophic pyramid in the steppes areas, their reduced number of specimens impacts other protected species such as *Aguila heliaca, Clanga pomarina* or *Mustela eversmanii*, which have this rodent as the principal prey (4, 5, 8).

Taxonomically, the *Spermophilus* genus has 41 different species (3, 9). The morphological information on the external ear can be corroborated with some morphological and morphometric body assessments and used to differentiate the European ground squirrel from other close species (8). Because their social behaviour includes the production of loud sounds as an alarm against imminent danger, the ear and its associated structures need to be in perfect condition (10) to fulfil the defence or various adaptive functions.

Anatomically, the ear or the vestibulocochlear organ (*Organum vestibulocochleare*) is one of the organs of the special senses, which accommodates essential structures for hearing and balance (11–13). Located in the temporal bone area, the ear may be divided into three separate segments: external ear (*Auris externa*), middle ear (*Auris media*) and internal ear (*Auris interna*) (13–16). The first two segments have an important role in sound wave conduction, and the third segment is responsible for the transformation of sound vibration into nervous impulses (15) The elements responsible for sound transmission are the tympanic membrane (*Membrana tympani*) and a bony chain of auditory ossicles (*Ossicula auditus*) located in the middle ear cavity (17–19), partly in the tympanic cavity (*Cavum tympani*) and partly in the epitympanic recess (*Recessus epitympanicus*).

Due to its complex anatomy, ear morphology can inform phylogenetic studies in rodents (20). As a general observation, for mammalian species which spend a long period in subterranean burrows, the ear seems to display some morphological adaptive features such as a well-developed tympanic bulla, a large eardrum, an absent *pars flaccida*, undeveloped or absent *tensor tympani* or stapedial muscle, undeveloped middle ear ligaments and a well-developed footplate (21, 22).

Since the *Spermophilus citellus* is listed as a ‘threatened’ species, the most recent research which involved this rodent was focused on different conservation programmes (5).

To the best of our knowledge, the morphological information related to the studied specimens is quite rare and is focused on the cardiovascular system or splanchnology (7, 23) and forelimb anatomy (2). A very complex morphological description of the auditory ossicles is available for American sciurids, lacking morphometrical data and reference to European specimens (24) while for another related family (Gliridae), the literature describes morphological data and some measurements related to the stapedial footplate (25).

The present study aims to provide accurate morphological and detailed morphometric information, which will add supplementary data related to middle ear anatomy in a threatened species. As per our knowledge, this is one of the few studies focused on the auditory ossicle chain in European ground squirrels (*Spermophilus citellus*), and it can serve as an addition for new morphological studies on other related species.

## Materials and methods

### Sample collection and image processing technique

10 dry skulls of *Spermophilus citellus,* part of an anatomical collection of Anatomy Discipline of the Faculty of Veterinary Medicine from Belgrade (Republic of Serbia) have been provided and investigated by our team. The bones are part of a comparative collection existing at the Department of Anatomy. The most recent specimens originate from road kills or cases found at the Clinics. The use of such comparative collection supports ethical research practices, promoting the responsible use of available sources instead of experimental animals.

After the identification of the bony part of the ear canal and the tympanic bulla, all the specimens were dissected in the Anatomy Lab of the Faculty of Veterinary Medicine Cluj-Napoca, Romania. Using an electric circular saw, serial fine cuts have been performed, and the lateral wall of the tympanic cavity has been removed for both right and left ear structures. To facilitate an accurate observation of the tympanic cavity, the bony splinters have been removed with an air jet. Since the biological materials consisted of dried skulls, only the bony structures housed in the tympanic cavity have been observed.

The auditory ossicles were studied *in situ* and immediately after having been removed for a specific examination. For images, we used an Olympus SZX7 dissection microscope (Olympus Surgical Technologies America) with an incorporated DP27 camera. The system was associated with the cellSens Standard Imaging Software (Olympus Surgical Technologies America). Later, for fine contrast and background fixes, we used Adobe Photoshop programme (San Jose, CA 95110-2704). The morphometric analysis used mostly ImageJ 1.46 software (26) and additional add-ons for measurements.

The present study falls under the 01-218 decision of the Committee of Ethics of the University of Belgrade and 353-01-752/2008-03 approval of the Ministry of Environment of the Republic of Serbia.

### Morphometric aspects and measurements

Measurements of the ossicles were attained following a standardised and widely accepted systematic approach, which previous researchers have well-described in otology studies of humans and other primates (27) (Tables 1–3). Special emphasis is placed on some measurements that are considered to be functional lengths (28, 29) (as in the case of the malleus and incus) that may be used in calculating the lever ratios in the middle ear to elucidate and describe the mechanical advantage associated with the lever system in the ear.

**Table 1:** Measurement protocol and indices for the malleus (28).

Measurement	Reference points
X axis	Midpoint of the minimum neck width—the most noticeable point along the top of the head
Y axis	Most inferior point of the short process and the manubrium
1 total length	Tip of the manubrium to the top of the head
2 manubrium (functional) length	Tip of the short process to the tip of the manubrium following Y axis
3 manubrium M-L thickness	M-L thickness of the manubrium at mid manubrium length, perpendicular to Y axis
4 manubrium arc depth	Maximum depth of the curvature of the arc of the manubrium, following Y axis
5 corpus length	Tip of the head to the lower border of the manubrium following X axis
6 neck width	Anterior and posterior borders of the neck
7 S-L head width	Maximum distance between 2 parallel lines marking the widest points of the margin of the head, taken following the X axis
8 angle between axes	X-Y angle
manubrium/length index	(Manubrium length/total length) * 100
manubrium robusticity index	(Manubrium ML thickness/corpus length) * 100
manubrium/corpus index	(Manubrium length/corpus length) * 100
corpus/length index	(Corpus length/total length) * 100

**Table 2:** Measurement protocol and indices for the incus (28).

Measurement	Reference points
X axis	Line that joins the most salient point along the anterior portion of the superior border of the body
Y axis	Line that joins the tip of the long process to the most salient point along the superior border of the body
Z axis	Line joining the tip of the long process to the most external point along the margin of the anterior facet
9 Short process length	Maximum distance from the tip of the short process to the most salient point along the anterior portion of the superior border of the body, following X axis
10 Long process length	Maximum distance from the tip of the long process to the most salient point along the superior border of the body
11 Functional length of the long process	Perpendicular distance from the Z axis (rotational axis) to the tip of the long process
13 Articular facet height	Max height of the articular facet with the bone oriented along the rotational axis
14 Angle between the axes	Angle formed by the X and Y axes
15 Interprocess length	Maximum distance between the most salient points along the superior margin of the short process and the tip of the long process
16 Interprocess arc depth	Maximum depth of the curvature between the short and long process tips
Incudal index	9/10 * 100
Long process index	11/10 * 100
Relative articular facet height	13/10 * 100

**Table 3:** Measurement protocol and indices for the stapes (28).

Measurement	Reference points
X axis	Line joining the antero-superior corner of the footplate and the tip of the head
Y axis	Line joining the posterior-superior corner of the footplate and the tip of the head
Z axis	Line joining the most inferior points along the footplate margin anteriorly and posteriorly
19 Total height of the stapes	Maximum height from the lower margin of the footplate to the tip of the head perpendicular to the Z axis
20 Head height	Minimum distance between the superior margin of the obturator foramen and the top of the head, taken perpendicular to the Z axis
21 Obturator foramen height	Maximum height of the obturator foramen taken perpendicular to the Z axis
22 Obturator foramen width	Maximum width of the obturator foramen taken parallel to the Z axis
23 Maximum width of the crura	Maximum width across the anterior and posterior crurae, taken on the external aspect and parallel to the Z axis
24 Posterior crus length	Maximum distance from the posterior-superior corner of the footplate to the tip of the head, following Y axis
25 Posterior crus arc depth	Maximum depth of the curvature of the posterior crus taken parallel to the Y axis
26 Anterior crus length	Maximum distance from the anterio-superior corner of the footplate to the tip of the head following X axis
27 Anterior crus arc depth	Maximum depth of the curvature of the anterior crus taken parallel to the X axis
28 Angle A	Angle between the anterior and posterior crurae or between the X and Y axes
29 Angle B	Angle between the anterior crus and the footplate or between the X and Z axes
30 Angle C	Angle between the posterior crus and the footplate between Y and Z axes
31 Footplate length	Maximum length of the footplate
32 Footplate width	Maximum width of the footplate
33 Footplate area	Measured area of the footplate
Relative head height	20/19 * 100
Obturator foramen index	21/22 * 100
Footplate index	31/32 * 100
Crural index	36/24 * 100

For some area calculation of the stapedial footplate, the elliptic tool from the ImageJ application was used to overlap the tool onto a digital image and thus automatically calculate the surface of the measured surface.

The standardised measurements and measurement protocols have been previously described for each of the ossicles. All measurements were taken by one operator using the same method of measurement and calibration in ImageJ and the same calibration scale over a short period, and all photographs were taken by another operator (to reduce biases). Measurements were taken from a series of images and different angulations. This approach made some measurements not possible due to the different presentation of an anatomical piece. Data were recorded by hand and then imported into a Google calculation sheet (allowing the direct calculation of indices). Data was recorded separately using the identification of an individual. Basic statistical processing was carried out with Google Sheets’ simple statistics feature, the XL Miner Analysis Toolpack add-on 2023 [FrontlineSolvers—www.solver.com (accessed on December 2024)], and the XLSTAT Cloud app.

## Results

**Figure 1:**
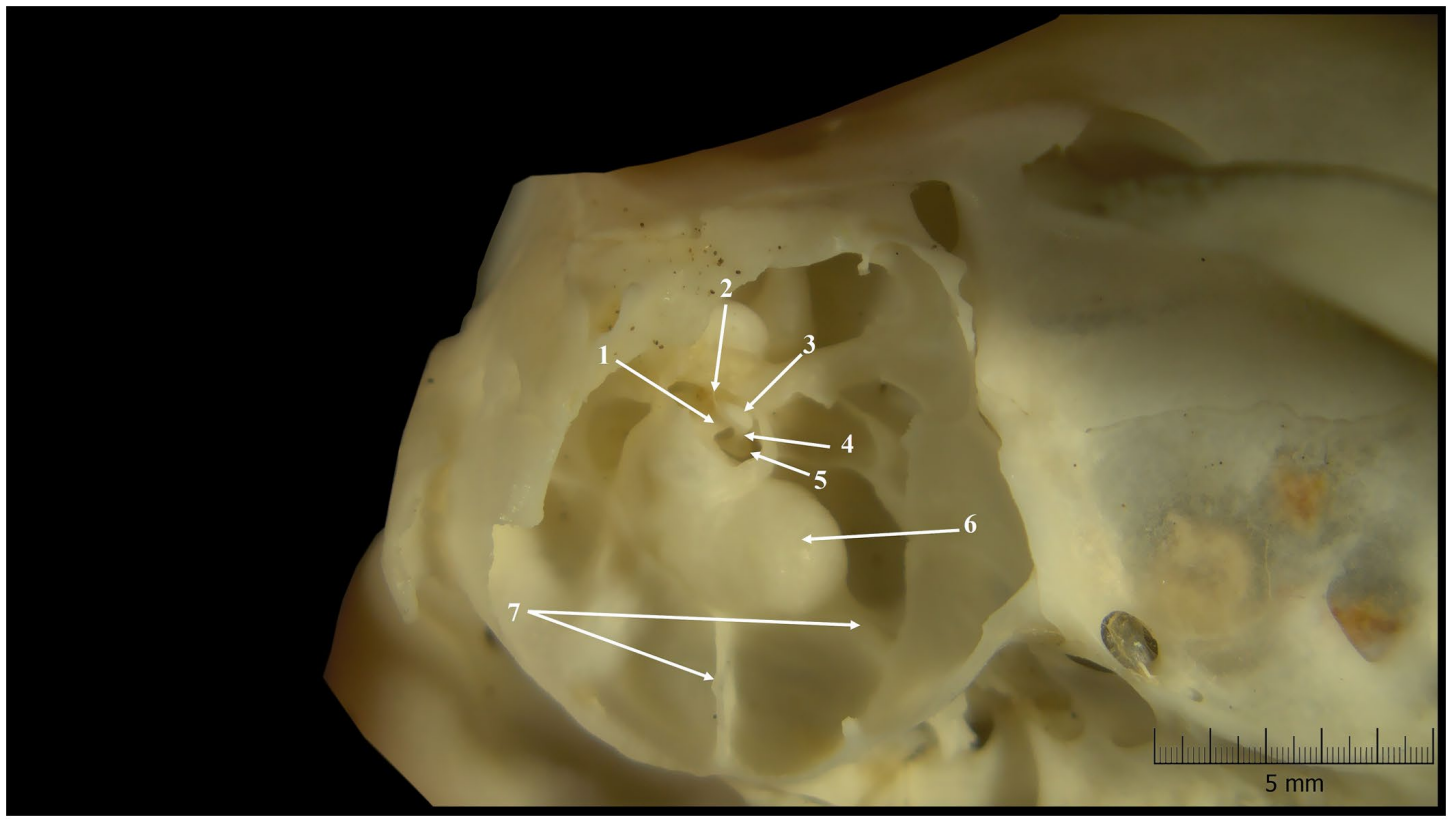
Tympanic cavity-inner features (lateral-ventral perspective). 1. Bony tube 2. Insertion tendon of stapedius muscle (dried); 3. Head of the stapes; 4. Rostral crus; 5. Base of the stapes; 6. Cochlear promontorium; 7. Bony septa.

The external examination of the European ground squirrel skulls allowed us to observe the external features of the middle ear. The first observed element was a short bony segment of the external ear canal. A wide tympanic ring marked the connection between this segment and the tympanic cavity. Looking through this orifice, the first visible element is the handle of the malleus, a segment which, normally, on fresh specimens, is in direct contact with the tympanic membrane. Even if the bony area which surrounds the ear canal showed a high density, the ventral wall of the tympanic cavity was very thin and almost transparent (Figure 1). This transparency facilitated the observation of a few bony septa, which have divided the hypotympanic area into smaller compartments. On their caudal area, the tympanic bullae have been bordered by small paracondylar processes (Figure 1).

The interior of the tympanic cavity allowed us to identify three different compartments. In a dorso-ventral direction, the most dorsal was the epitympanic recess, and the most ventral was the hypotympanum, which contained within the tympanic bulla (Figure 1). The middle compartment was the mesotympanum. Each of them accommodated distinctive elements of the middle ear.

In the investigated specimens, the mesotympanic compartment allowed the identification of a well-developed cochlear promontorium. A few bony septa could be observed between the caudal-ventral walls of the cochlear promontorium and the marginal walls of the middle ear. These septa have compartmentalized the hypotympanum in small sub-cavities.

The biggest and with the most lateral disposition, the malleus, was the first auditory ossicle which could be observed. Viewing the malleus macroscopically, it showed the classical aspect and facilitated the identification of three different segments: the head (*Caput mallei*), the neck (*Collum mallei*) and the handle (*Manubrium mallei*). The *caput mallei* have a rounded tuberous aspect and send rostrally a pointed anterior process (*Processus rostralis*).

This process has a triangular shape and connects the malleus with the tympanic ring. The lateral surface of the head showed a smooth aspect, proof that the suspensory ligament of the malleus and the lateral suspensory ligament of the malleus, structures which connect the malleus to the bony walls of the middle ear, are not well developed. The opposite surface bears a large V-shaped articular surface, surrounded by bony margins, for articulation with the second auditory bone, the incus.

**Figure 2:**
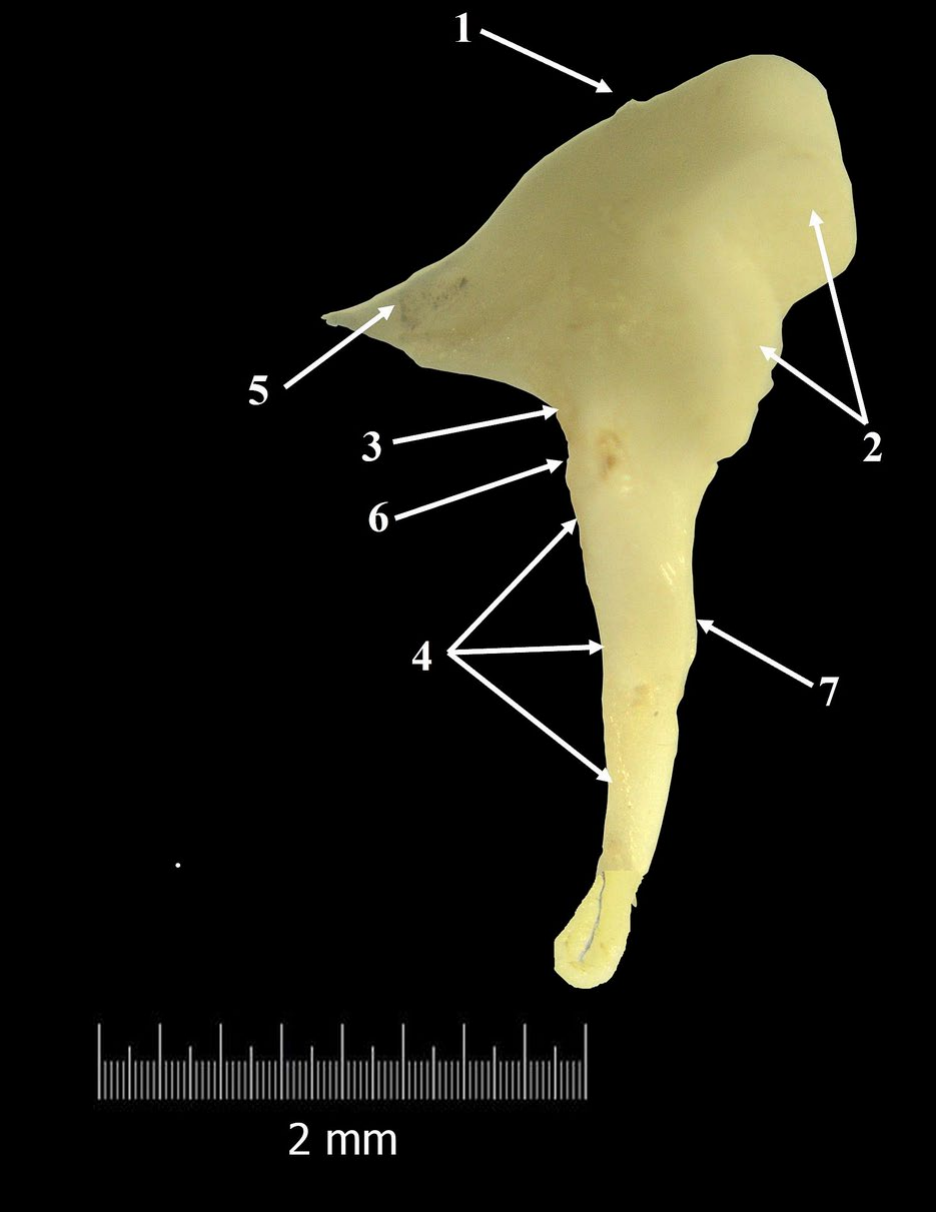
Malleus-macroscopical features (composite photograph). 1. Head of the malleus; 2. Articular surface; 3. Neck of the malleus; 4. Handle of the malleus; 5. Anterior process; 6. Lateral process; 7. Muscular process.

**Figure 3:**
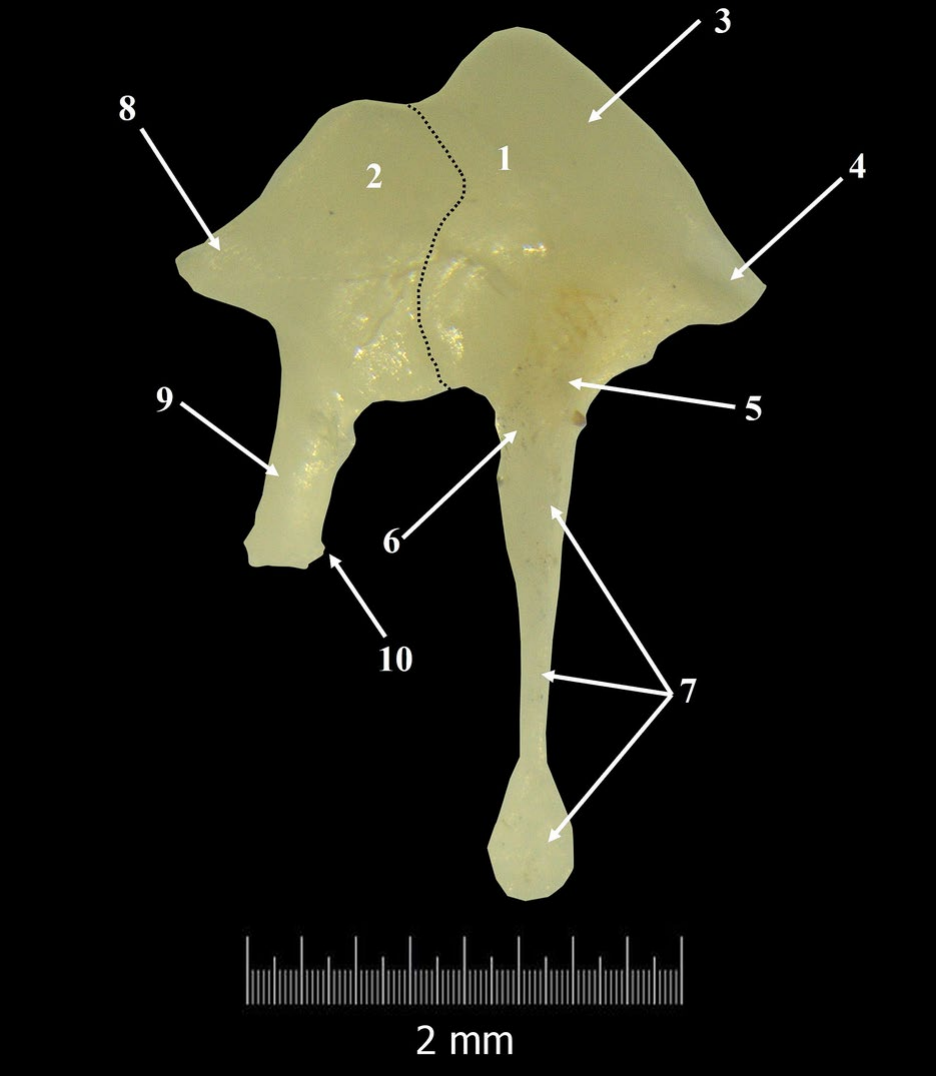
Incudo-malleal complex. 1. Malleus; 2. Incus; 3. Malleal head; 4. Anterior process; 5. Neck of the malleus; 6. Lateral process; 7. Handle of the malleus; 8. Short crus of the incus; 9. Long crus; 10. Lenticular process; Dotted line-articulation line between malleus and incus.

The next segment, *collum mallei* (Figures 2, 3), is very short, almost indistinctive in all studied specimens, and continues ventrally from the malleal head (Table 4). Due to its length in *Spermophilus citellus,* this segment does not bear any bony process. Distally, the limit between the neck of the malleus and the handle of the malleus is marked by a small prominence, the lateral process (*Processus lateralis*) (Figures 2, 3), located on the lateral surface of the malleus. The manubrium mallei has an almost perpendicular direction on the anatomical axis of rotation of the malleo-incudal joint. Near the malleal neck, in a cross-section, the manubrium has a triangular profile, but this aspect changes distally, where this segment shows a spatulate shape. The medial surface of this segment allowed us to identify, approximately in its middle area, a small bony prominence, which is the insertion point for the tensor tympani muscle (*Processus muscularis*). In all the specimens studied, the malleus and incus were individual bones.

**Table 4:** Summary of the dimensional variations for malleus in the studied *Spermophilus citellus* specimens.

Measurement number	Mean values (mm)	Standard deviation
1 (*n* = 9)	3.52	0.394
2 (*n* = 6)	2.56	0.404
3 (*n* = 8)	0.43	0.066
5 (*n* = 9)	2.20	0.301
6 (*n* = 8)	1.24	0.147
7 (*n* = 8)	1.00	0.174
8 (*n* = 8)	73.17	2.537

**Figure 4:**
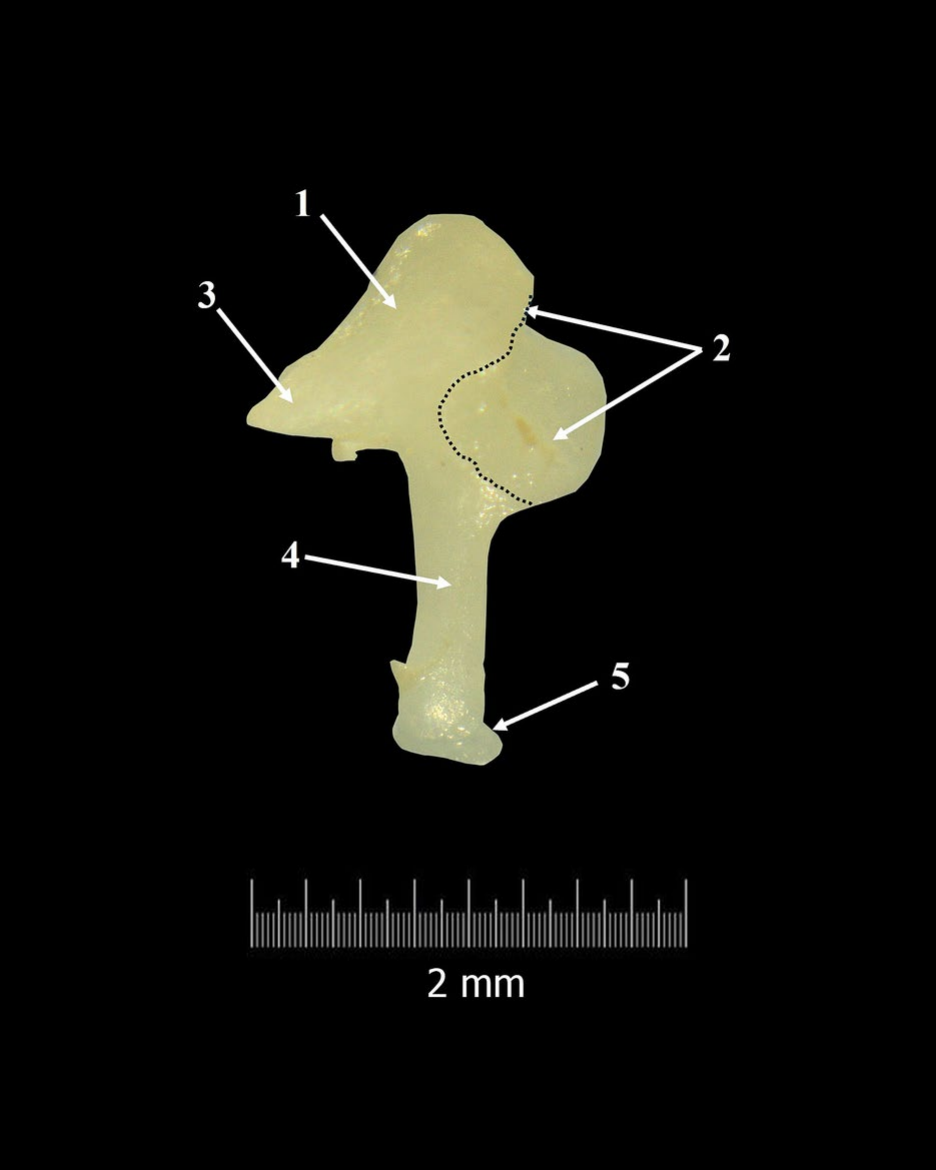
Incus-macroscopical features. 1. Body of the incus; 2. Articular surface; 3. Short crus; 4. Long crus; 5. Lenticular process; Dotted line-margin of incudal articular surface.

In a latero-medial direction, the second auditory ossicle was the incus (Figures 3, 4). It is smaller than the malleus and possesses a rectangular body (*Corpus incudis*) and two processes. This ossicle follows the classical description, being like a biradicular molar tooth.

Overall, the incudal body has a rectangular shape and is very short (Table 5), the dorso-ventral diameter being longer than the latero-medial one. The lateral extremity of the incudal body bears a large articular surface, the negative of the malleolar articular surface, which will be part of the incudo-malleolar joint (*Articulatio incundomallearis*) (Figure 3). It is very important to mention that the margins of the articular surface ventrally almost touch the origin of the long process of the incus.

**Table 5:** Summary of the dimensional variations for incus in *Spermophilus citellus* specimens.

Measurement number	Mean values (mm)	Standard deviation
19 (*n* = 3)	1.435	0.0212
20 (*n* = 3)	0.35	0.0141
21 (*n* = 3)	0.86	0.028
22 (*n* = 3)	0.73	0.0707
23 (*n* = 5)	1.005	0.0354
24 (*n* = 3)	1.34	0.0283
25 (*n* = 3)	0.19	0.0141
26 (*n* = 3)	1.265	0.04895
27 (*n* = 3)	0.135	0.0071
28 (*n* = 4)	60	5.65
29 (*n* = 4)	61.5	3.53
30 (*n* = 3)	57	4.24
31 (*n* = 4)	1.542	0.086
32 (*n* = 4)	0.4967	0.0306
33 (*n* = 4)	0.6573	0.1129

In a caudo-medial direction, the incudal body continues with two unequal processes. The dorsal border of the incus continues caudally with a short conical-shaped process known as the short crus (*Crus breve*) (Figures 3, 4). Topographically, in *Spermophilus citellus,* this process lines up with the rostral process of the malleus.

In a ventral direction, the second process, *crus longum* (Figures 3, 4), is much longer than the first one (*crus breve*) (Table 6). More than that, if the malleo-incudal joint is still intact we can see that the long process of the incus has a parallel direction with the malleus handle, both of them perpendicular to the anatomical axis of rotation of the malleo-incal joint.

**Table 6:** Summary of the dimensional variations for incus in *Spermophilus citellus* specimens.

Measurement number	Mean values (mm)	Standard deviation
9 (*n* = 8)	1.3917	0.076
10 (*n* = 5)	2.345	0.0706
11 (*n* = 5)	1.5183	0.0823
13 (*n* = 6)	0.9483	0.0943
14 (*n* = 8)	49.89	2.853
15 (*n* = 4)	1.747	0.016
16 (*n* = 4)	0.5117	0.0286

The distal third of the long process bends medially and ends with an ellipsoidal structure, the lenticular process (*Processus lenticularis*) (Figures 3, 4). The bent segment, known as the bony pedicle, was short in *Spermophilus citellus,* and wide.

**Figure 5:**
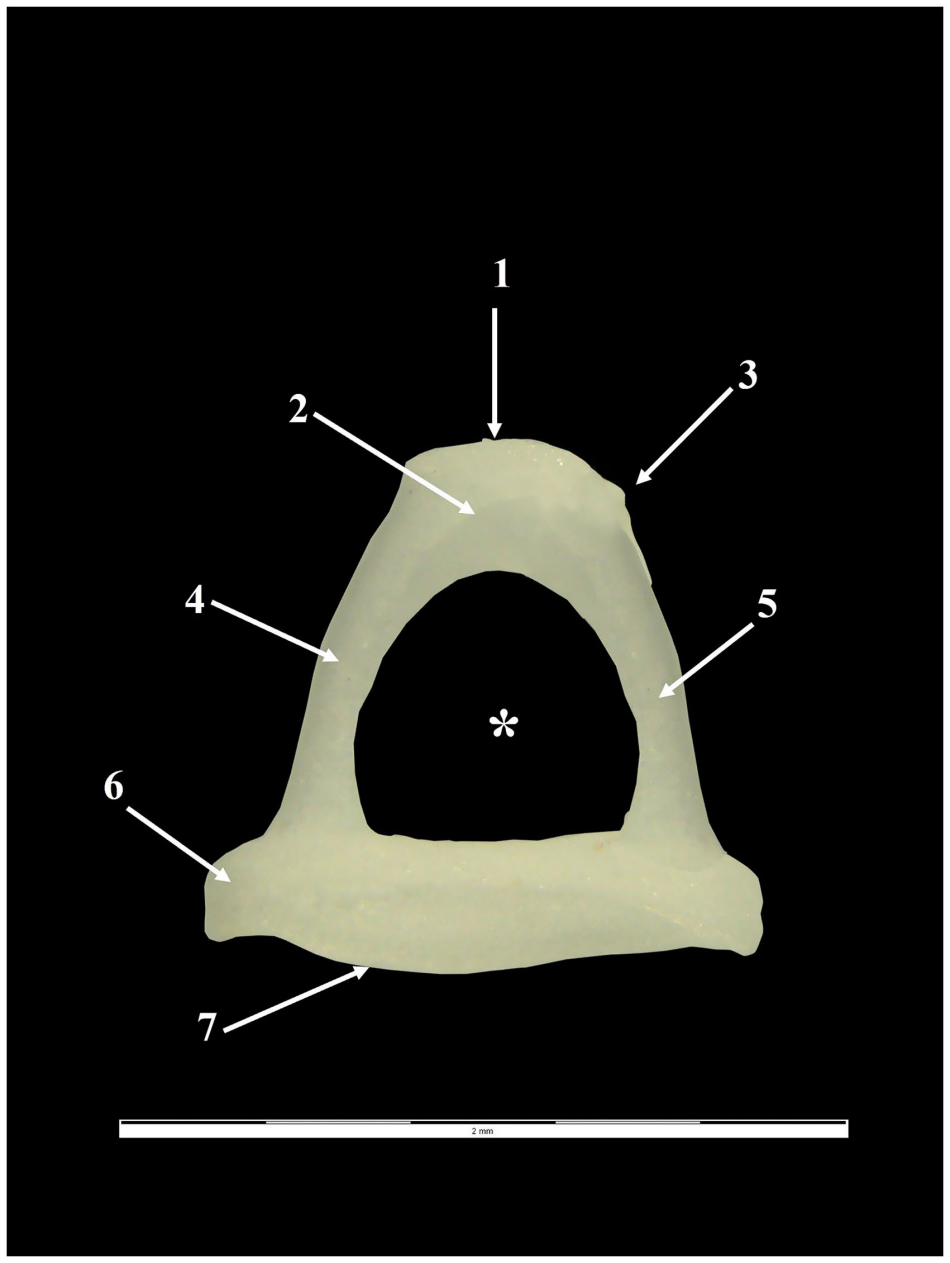
Stapes-macroscopic features. 1. Articular surface of the stapes; 2. Head of the stapes; 3. Muscular process of the stapes; 4. Rostral crus; 5. Caudal crus; 6. Footplate; 7. Vestibular surface of the base of the stapes; ^*^Intercrural foramen.

The most medial auditory ossicle was the stapes (*Stapes*) (Figure 5), and it was located between the lenticular process laterally and the oval window (*Fenestra vestibuli*) medially. The macroscopic features of this auditory bone show us that his bone is the smallest one of the studied specimens (Figure 5). Specific for the European ground squirrel, the mesotympanic area builds a supplementary cavity which surrounds and protects the stapes. This cavity has a dorso-caudal position in respect to the cochlear promontorium and its caudal wall sends cranially a bony tube which passes the stapedial intercrural space. This bony tube seems to protect, in living animals, the stapedial artery (*Arteria stapedis*).

With a mediolateral and ventral-cranial disposition, the stapes has a triangular shape in all studied specimens. Anatomically, it allowed us to identify the head of the stapes (*Caput stapedis*) with the most lateral position, and the base of the stapes (*Basis stapedis*) with the most medial position. The connection between the previously mentioned structures was realised by two bony columns, the stapedial crurae (*Crus rostrale* and *Crus caudale*) (Figure 5).

The stapedial head is very short (Table 7) and covered laterally by an ellipsoidal articular surface for articulation with the lenticular process. Its long diameter seems twice that of the short diameter. Caudo-laterally to the articular surface, a very thin bony tuberosity was observed for all specimens. This is the muscular process of the stapes, which serves as the insertion point for the distal tendon of the stapedius muscle (*M. stapedius*).

**Table 7:** Variance indices of the malleus in *Spermophilus citellus.*

Index malleus	Value (mean +/− SD)
Manubrium/length index	70.82 ± 2.462
Manubrium robusticity index	19.57 ± 1.739
Manubrium/corpus index	116.2 ± −3.134
Length index	63.33 ± 2.192

The stapedial footplate seems relatively large (Table 7) and shows an elliptical shape, with a double vertical diameter compared with the horizontal one. Its circumference is surrounded by an evident bony ridge. Between this structure and the edges of the oval window, there is a space. The mentioned gap, in live animals, is covered by a wide annular ligament (*Ligamentum anulare stapedis*). The tympanic surface of the footplate is concave and has an excavated aspect, and the vestibular side is convex and protrudes into the inner ear.

The stapedial crurae connect the stapedial head with the stapedial base and are more or less different. They have a similar diameter, but the caudal crus is straight while the rostral one shows a reduced ventrocranial convexity (Table 8). This aspect makes the rostral crus a little bit longer than the caudal crus. The internal aspect of both crura is excavated, and they surround a large intercrural foramen with a circular shape. It is important to note that it has a complete bony canal, which in fresh specimens accommodates the stapedial artery.

**Table 8:** Variance indices of the incus in *Spermophilus citellus.*

Index incus	Value (mean +/− SD)
Incudal index	59.4645 ± 3.7096
Long process index	64.8483 ± 4.901-
Relative articular facet height	40.3879 ± 3.1247

## Discussion

The data described here shows very similar structures of *Spermophillus* to those of other squirrels and their close relatives, with some minor differences noted in the following lines.

A description of an inflated tympanic bulla has been provided for non-gliding sciurids such as *Sciurus hudsonicus, Sciurus niger rufiventer, Callosciurus flavimanus* and *Nannosciurus* (30). As in *Spermophilus citellus*, in the named species, the tympanic bulla allowed identification of bony septa which started at the level of the cochlea and radiated by reaching the sulcus tympanicus to create what literature sources describe as the specific intrabullar septal pattern (31). A well-developed tympanic cavity, more or less similar to that reported for the studied specimen was also mentioned in a few rodent species of several families such as: the mole-rats (32), degu (*Octodon degu*), chinchilla (*Chinchilla lanigera*), guinea pig (*Cavia porcellus*) (13, 22), gerbil (*Desmodillus auricularis*), Setzer’s hairy-footed gerbil (*Gerbillurus setzeri*), kangaroo-rats (*Dipodomys phillipsii*) (33), tuco-tuco (*Ctenomys latro*) (34), same for the elephant shrew (*Elephantulus rupestris, Macroscelides flavicaudatus*) (an insectivourous of Macroscelididae family) and fossorial species as an important adaptation to the low-frequencies sound conduction.

A relatively small middle ear cavity, characterised by the lack of bony septa, has been described for rats (22). As in some members of the Sciuridae family—*Glaucomys volans* and *Citellus t. tridecemlineatus* (30), in our specimens, we could observe the presence of the paraoccipital processes caudal to the tympanic bulla.

Even if the tympanic cavity is well-developed and the tympanic bulla is significant in size, in the European ground squirrel, we did not observe the direct communication between the right and left middle ear cavities. Similar situations have been reported in *Octodon degus* (family Octodontidae) (22), *Meriones, Desmodillus, Gerbillurus* (family Muridae) (35–37), *Neurotrichus, Parascalops* and *Condylura* (members of Talpidae family) (38) and non-rodents such as *Macroscelides,* where the tympanic cavities maintained a separate character. Different features were reported in other talpids such as *Scapanus* and *Parascaptor,* where trans basisphenoid connections have been providing direct communications between the right and left middle ears (38).

Macroscopic features of the auditory ossicles described by us in *Spermophilus citellus* are consistent with the anatomical records reported by other authors in freely mobile types of auditory ossicles (35–37, 39, 40). According to several sources (21, 35, 41), this type of auditory ossicles is common for a large number of subterranean mammal species and is marked by a weak articulation of the auditory bones with the bony elements of the tympanic cavity and poorly developed or even absent middle ear muscles, a large stapedial base and also a wide tympanic membrane that presents only the pars tensa *(Pars tensa).* The large tympanic bulla, together with the above-mentioned characteristics of the auditory bones, has been proven experimentally to improve low-frequency sound transmission in some caviomorphs (42).

Even if the rodentia order is huge, the more complete otology studies are focused on guinea pigs, rats and hamsters (13, 43), dormice such as hazel dormouse (*Muscardinus avellanarius*), edible dormice (*Glis glis*) and the Eastern grey squirrel (*Sciurus carolinensis*) (25, 33, 44). Like the reported data for *Rattus norvegicus* (22), *Eospalax fontanierii, Spalax galili, Spalax golani, Spalax judaei* and *Tachyoryctes splendens* (32) in *Spermophilus citellus,* the first two auditory ossicles were completely separable. The same features have not been reported for *Ctenomys sociabilis* (45), *Heterocephalus glaber* (35, 37), *Octodon degus* (22), *Chinchilla lanigera* (13, 22, 46), *Cavia porcellus* (46) and *Cuniculus paca* (47), where the authors reported fused malleus and incus (48), a rare feature that is listed by literature sources as the Ctenohystrica type of auditory apparatus. In humans, the malleus and incus are two individual bones (49–51), and the malleo-incal joint maintains its cartilaginous characteristics throughout life (13, 52).

A synostosed malleo-incudal complex is related, in humans and mice, to congenital deafness (53, 54).

In accordance with the reported data by Segall (30), in *Spermophilus citellus,* the macroscopic features allow us to describe it as a specialised type of malleus. As in our specimen, this type of malleus has a dome-shaped malleal head, and a saddle-shaped articular surface. Similar to his description, in our specimens, we reported a very short, almost indinstinctive neck of malle.

Similar features of the first auditory bone have been also reported in non-gliding sciurids as *Sciurus hudsonicus, Sciurus niger rufiventer*, and *Citellus t. tridecemlineatus* (30). The overall aspect of the malleus in the European ground squirrel also has very similar characteristics to the reported data in *Meriones, Desmodillus* (family Muridae) and *Jaculus* (family Dipodidae), whose malleus is characterised by a large malleal head, lack of the orbicular apophysis and a perpendicular manubrium on the rotational axis (35, 37).

Even if in other species all three malleal processes have been reported as bony expansions of the malleal neck (11, 16), in our specimens, the size of this segment made the identification and individualisation of these processes very difficult. Like anatomical features reported in other rodents (24, 30, 35, 45), primates (16, 55–57) and some carnivores (58), the muscular process of *Spermophilus citellus* was part of manubrium mallei and served as an insertion point for the tensor tympani muscle. Since we used dried skulls, we were unable to assess the presence of the tensor tympani muscle within the middle ear of the studied species. This structure has been confirmed in previous studies, which involved the chorda tympani nerve, which in *Spermophilus citellus* shows a hypotensoric course (20).

This muscle is present, but small in microtype species such as *Neurotrichus, Condylura, Parascalops* and is absent in freely mobile type species such as *Talpa, Scalopus, Scapanus, Parascaptor* (family Talpidae) (38). The anterior process, described by Cockerell et al. (24) as *processus gracilis,* in our investigated specimens had a triangular shape and is a rostral continuation of the malleal head. The third process of the malleus, the lateral one was very reduced. Similar features have been reported by Mason et al. (32) for three species of rodents of the Spalacidae family, *Tachyoryctes, Eospalax, and Spalax golani* or for a few non-gliding sciurids (30). Last one, describe this process as the processus brevis of the malleus. A well-developed lateral process has been described in *Talpa europaea* (Talpidae family) (38).

The second auditory ossicle in European ground squirrels shows the classical morphology described previously in the investigated literature sources (11, 35–37, 59). Similar to other members of the Sciuridae family, such as *Sciurus hudsonicus, Sciurus niger rufiventer, Nannosciurus, Citellus t. tridecemlineatus* (30), *Talpa, Scalopus and Scapanus* (family Talpidae) (38) in our specimens, the incus showed a well-developed body, a conical short process and a much longer long process. The same authors have reported that in other talpids as *Neurotrichus, Parascalops* and *Condylura*, the incus is very reduced in comparison with the malleus (38).

In the present study, the short process of the incus was disposed in an opposite location compared with the anterior process of the malleus, being part of the anatomical axis of rotation (39). Because in the studied specimens, between the two processes of the incus (the Z and X axis) we noticed a relative right angle, we can tell that the long process of the incus is perpendicular to the anatomical axis of rotation. Similar aspects have been observed in some small desert mammals (*Meriones, Desmodillus* of the Muridae family *and Jaculus* of the Dipodidae family) (33) and chinchilla (13, 22, 60). In *Spermophilus citellus,* the distal end of this process slightly curves terminally. This sector continues with the lenticular process, with no diameter change at the curvature level.

As in our specimens, in some species of the Dipodidae family such as *Jaculus jaculus* and *Jaculus orientalis,* the connection between the distal end of the long process of the incus and the lenticular apophysis is realised by a narrow pedicle (35, 37). Compared with our data, in *Jaculus,* the authors reported a long pedicle. Similar to the data reported by other authors (61), in cats, in *Spermophilus citellus,* this pedicle represents a point of high flexibility, which allows more movement than the incudostapedial joint itself.

As reported in the literature, the last auditory ossicle, the stapes is the smallest one and fills the gap between the lenticular process and the oval window (11, 16, 38, 59).

According to morphological characterisation provided by Segall (30), in *Spermophilus citellus,* we can report a specialised type of stapes. In our specimen, this bone had an elliptical footplate, the intercrural foramen was crossed by a bony canal which encloses the stapedial artery and more than that, according to our measurements, the stapedial ratio is 2.9 (Table 9).

**Table 9:** Dimensions and indices calculated for stapes.

Species/measurement	Stapedial footplate length (mm) (31)	Stapedial footplate width (mm) (32)	Footplate area (mm^2^) (33)	Footplate index/stapedial ratio
*Trichosurus vulpecula* (30)				170^*^
*Phalanger lemuroides* (30)				200^*^
*Sus scrofa domestica (embrional)*	1.82	1.08	0.7125^*^	173.148
*Ratus norvegicus* (57)	0.81^*^	0.46^*^	0.289^*^	176
*Capra hircus* (54)	2.29	1.26	2.167	178.9
*Cavia porcellus* (76)	1.44	0.66	0.79	208.69
*Chlorocebus sabaeus* (16)	1.68	0.775	0.959	214.29
*Ovis aries* (77, 78)	2.03	0.93	1.54	218.2
*Homo sapiens* (76, 78, 79)	2.81^*^	1.285^*^	2.92^*^	218.31
*Anomalurus fraseri* (30)				220^*^
*Hylopeies nigripes* (30)				220^*^
*Schoinobates volans volans* (30)				220^*^
*Pan troglodytes* (28)	2.7	1.22	2.72	221.31
*Gorilla gorilla* (28)	2.78	1.23	2.82	226.01
*Glis glis* (30)				250^*^
*Chinchilla lanigera* (13, 80)	2.09	0.83	1.307	251.8
*Sciuridae* (30)				265^*^
*Sciurus hudsonicus, Sciurus niger rufiventer* (30)				270^*^
*Muscardinus avelanarius* (30)				287^*^
*Spermophilus citellus*	1.542	0.4967	1.69	299.35
*Callosciurus flavimanus bouhotei* (30)				Approx. 300^*^
*Hamsters* (75)	0.89	0.24	0.169^*^	370^*^

^*^Values recalculated based on the basic metric data provided by authors or displayed as average in literature sources. For the values mentioned as a simple ratio, the conversion to the multiplied values was performed and illustrated in the present table.

Among mammals, this ossicle showed variable shapes (62–65). For our specimens, the stapes showed the typical aspect of this bone for placental mammals, having a triangular stirrup shape with two crura and a base (35, 37). This triangular shape of the stapes, reported in *Spermophilus citellus* has been described as a typical characteristic of third auditory ossicles of the gliding and non-gliding sciurids and in some insectivores *(Nasilio).* All named species possess a specialised type of stapes (30).

Similar to the reported data for *Octodon degus* (22) and some small desert mammals (35, 38), both stapedial crura of the studied specimens were hollowed on their inner side. As we reported for *Spermophilus citellus,* in some talpid species such as *Scalopus, Scapanus and Talpa* a small difference between the caudal and cranial crura has been mentioned, the caudal crus being (subjectively) straighter than the rostral one. Also, a longer rostral crus has been reported in New Zealand Rabbits (family Leporidae) (62, 66).

Even if the fact that we used dried skulls made it impossible for us to identify the stapedial muscle, the presence of the stapedial tubercule confirmed its presence. The same muscle has been confirmed as being present rodents of spalacid and murid species such as *Spalax ehrenbergi, Spalax galili, Spalax golani, Spalax judaei* (32), *Meriones, Desmodillus, Gerbillurus, Rattus norvegicus* (22, 59) or insectivorous, such as *Macroscelides/Elephantulus* (35, 37)and absent in *C. sociabilis* (45), *Octodon degus* (of Ctenomidae and Octodontidae families) (22), *Heterocephalus glaber, Bathyergus suillus, Cryptomys hottentotus, Fukomys micklemi, Georychus capensis* and *Heliophobius argenteocinereus* (members of the Bathyergidae family) (35, 37). The lack of stapedial muscle was confirmed by the absence of the stapedial muscular process.

The gap located between the marginal circumference of the stapedial base and the petrosal oval window has been reported previously in other rodents and looks to be one of the adaptations to improve low-frequency sound transmission (32, 35, 37).

Among rodents, the stapedial artery is inconstant. According to previously reported data, this artery passes the intercrural foramen and has a regressing character in mammals (25, 35, 37). In caviomorphs (parvorder Cavimorpha) (22), a bony process could be observed as passing the intercrural foramen. According to Parodi et al. (67) this bony tube is a remnant of the stapedial artery in rodents. The presence of a genuine bony canal for the stapedial artery reported by us in *Spermophilus citellus* (Figure 1), is similar to the reported data in *Callosciurus flavimanus bouhotei* and *Citellus t. tridecemlineatus* (30) but also in other non-rodent species such as *Macroscelides flavicaudatus* (35). This particular aspect of the tympanic cavity is reported as an adaptation to low-frequency sound transmission, which protects the auditory organ against low-frequency residual noise (39, 68).

**Figure 6:**
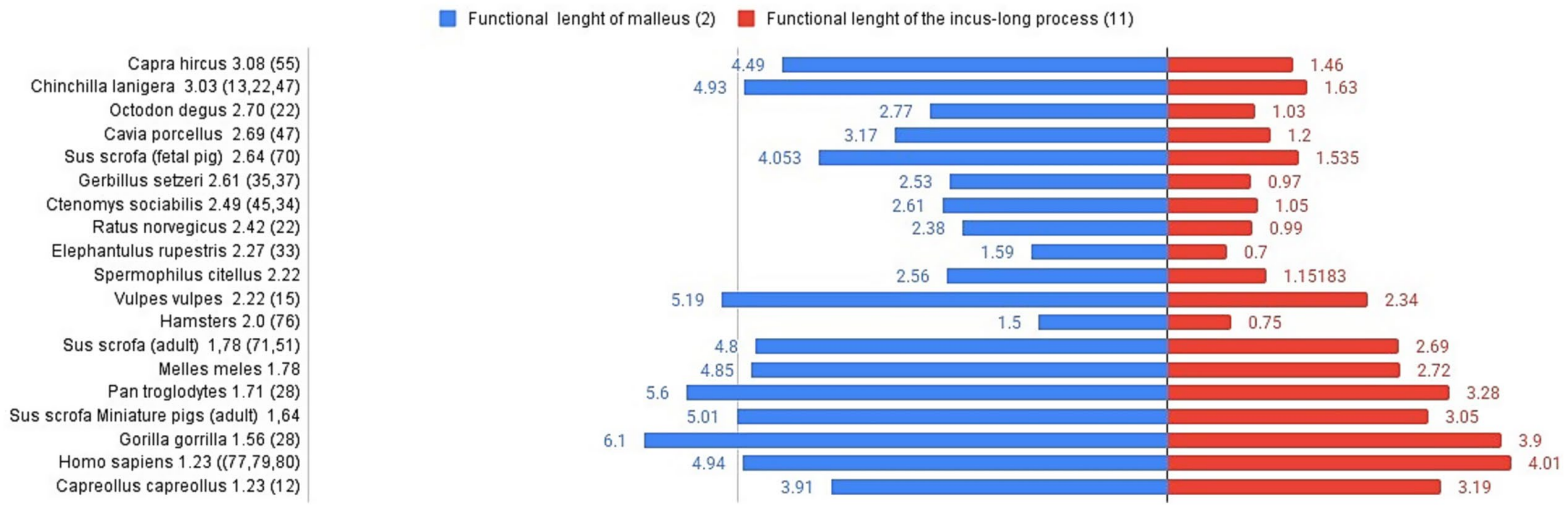
Graphical representation of malleus and incus (functional lengths) for different species. Lever ratio is listed in the left registry of the image in descending order. Functional length of malleus (mm)-left registry, functional length of the incus long process (mm)-right registry.

The lever ratio compares the functional lengths of the malleus (Table 6) and incus (Table 5) based on their rotational axis inside the tympanic cavity, adding a standard set of measurements that will characterise in detail the necessary measurements of the malleus and incus, based on the estimation of the axis of the malleo-incal joint. This assembly plays a key role in sound transmission, providing a mechanical advantage, thus being a relevant physiological variable in the modelling of the audition (64, 65) Still, other sources mention the fact that at low sound frequencies, the figures provided by these calculations are not necessarily accurate in terms of hearing abilities for the small differences among species (35). The functional length of the malleus (considered to be the manubrium length-measurement 2) and the length of the incus (considering the functional length of the long process-measurement 11) (Tables 5, 6) are the two reference values for the calculation of the lever ratio (Figure 6).

Comparative data available show some notable differences among species, despite their very different habitats (and the availability of literature data, also). In terms of absolute values, based on the available measurements in *Capra hircus* (54), foetal pigs (69), fox (*Vulpes vulpes*) (15) and even adult pigs or badgers (70) we can only assume that the value of the functional ratio keeps our investigated species very close to other rodents (35) (rat, gerbil, elephant shrew, hamster, guinea pig), whereas the different primate species (16, 28, 71–73) seem to be placed close to the roe deer (12), in the lower registry of values for this index. Another interesting fact is shown in the lower part of the graph’s registry, placing some primates and miniature swine (74) as species with a low lever ratio value.

Although several sources reveal measurements of the auditory ossicles for a multitude of species (domestic, wild, of different habitats), the attention seems to be drawn more towards malleus and incus (as functional indicators) as the stapes usually was targeted for oval areas and crus dimensions. The footplate is usually measured at its maximal length, while the width is rarely measured. From the available sources describing the ossicular anatomy and metrics, we managed to attempt a summing up of some values, as seen in Table 9.

**Figure 7:**
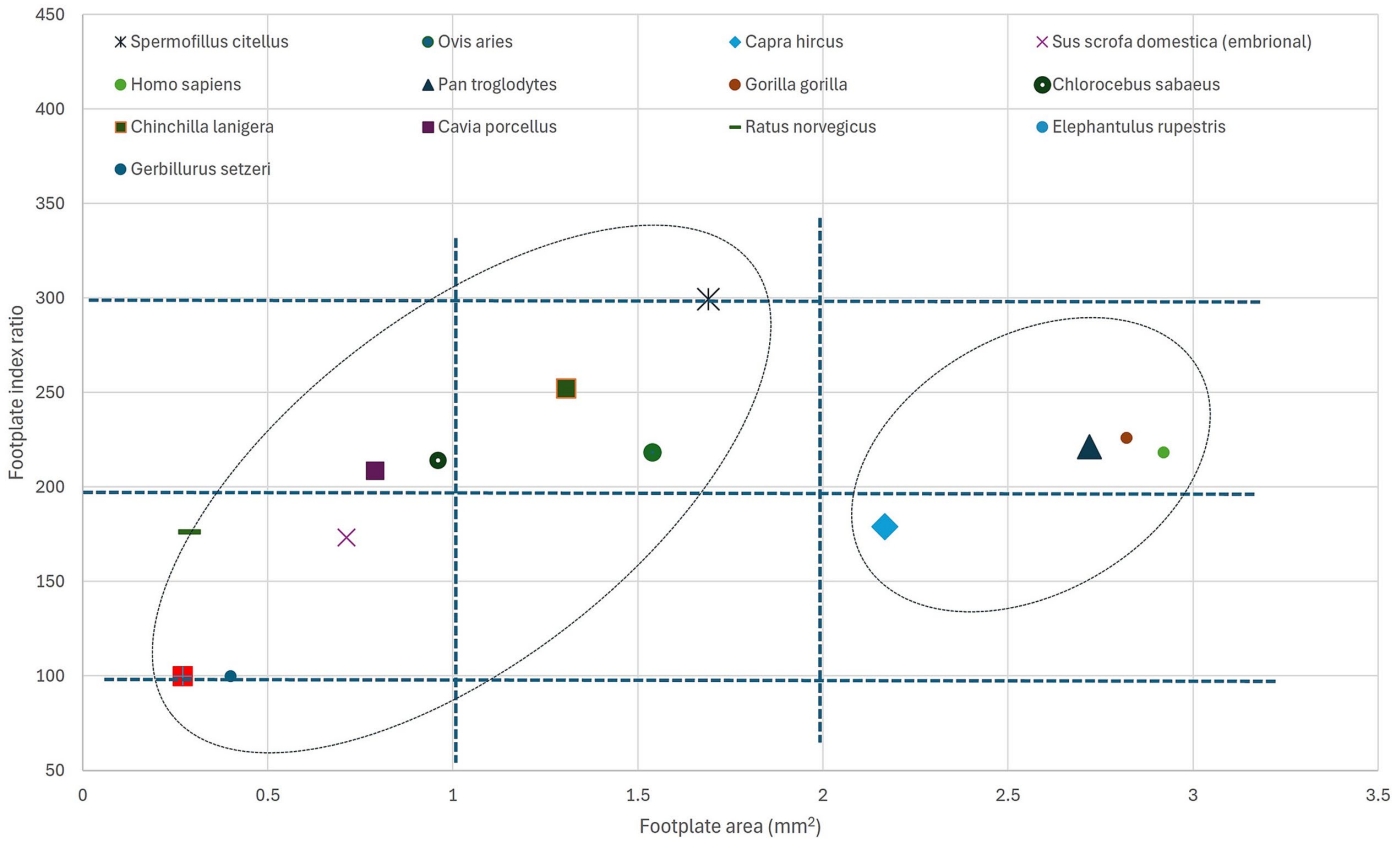
Graphical representation of the stapedial index and stapedial footplate area for different species. For the source data for each species, see Table 9. Ellipses point to the possible grouping of species.

The available data, still not completely comprehensive, as it does not cover a large array of species and habitats, shows an interesting distribution from the perspective of the stapedial footplate dimensions. In an attempt to represent the investigated species’ footplate features (Figure 7), pointing to the calculated area of the footplate and the footplate index (which might appear expressed either as a simple ratio between length and width of the footplate named ‘stapedial ratio’—as in Farr and Mason (25) or as a figure multiplied by 100), one can see the division of the groups of species in several quadrants (0–1 sq. cm, 1–2 sq. cm, more than 2 sq. cm for the footplate area) and the intervals for the index (under 200, 200–300 and higher than 300). The grouping of some primate species appears in the right registry of the graph, while most of the rodents (35) are in the left registry (with very high values for the index in the case of hamster (75) but low area values, indicating an elongated stapedial footplate). The investigated species, the ground squirrel, is placed in this graph in the middle registry, with a median value for the area of the footstapes with a high stapedial index (over 300 value).

The footplate index, as a base value, has been considered in anatomical and phylogenetic studies as an indicator of primitive mammalian character (25). As represented in Table 9, illustrating literature values (in a unified manner) of a more extended list of species, the index shows an interesting distribution of values. The lower values (below 2 or 200) seem to be associated with the non-specialised type of stapes (found also in marsupials, some Artiodactyla), while the available data for primates and some Caviidae show slightly increased values (a little above 2 or 200). Most of the indices above the value of 2.5 or 250 are associated with members of the Sciuromorpha suborder.

## Conclusion

The present study provides a complete set of morphological and anatomical data, which improves and increases the anatomical knowledge related to the *Spermophilus citellus.* All data confirm the general framing of the studied specimens into the family Sciuridae. With such a detailed description, the present study can represent a starting point for other future research in the field of ear anatomy. All the anatomical characteristics of the described structures characterise the middle ear in European ground squirrels as being adapted to low-frequency sound transmission. The multitude of metrical elements illustrated here completes the morphological image, and the addition of some more complex attempts in the characterisation of the lever ratio and metrics of the stapedial footplate opens several new directions towards a more complex investigation focused not only on the pure morphology of the middle ear but also on other elements associated with hearing and integrative functionalities.

## Data Availability

The raw data supporting the conclusions of this article will be made available by the authors, without undue reservation.
